# Regulatory Framework for Advanced Therapy Medicinal Products in Europe and United States

**DOI:** 10.3389/fphar.2019.00921

**Published:** 2019-08-30

**Authors:** Carolina Iglesias-Lopez, Antonia Agustí, Mercè Obach, Antonio Vallano

**Affiliations:** ^1^Department of Pharmacology, Therapeutics and Toxicology, Universitat Autònoma de Barcelona, Barcelona, Spain; ^2^Clinical Pharmacology Service, Vall d’Hebron University Hospital, Barcelona, Spain; ^3^Medicines Department, Catalan Healthcare Service, Barcelona, Spain; ^4^Pathology and Experimental Therapeutics Department, University of Barcelona, Barcelona, Spain

**Keywords:** genetic therapy, tissue engineering, cell- and tissue-based therapy, biological products, biological therapy, legislation and jurisprudence, United States Food and Drug Administration, Europe

## Abstract

Advanced therapy medicinal products (ATMPs) are a fast-growing field of innovative therapies. The European Union (EU) and the United States (US) are fostering their development. For both regions, ATMPs fall under the regulatory framework of biological products, which determines the legal basis for their development. Sub-classifications of advanced therapies are different between regions, while in EU, there are four major groups, i.e., gene therapy, somatic cell therapy, tissue-engineered therapies, and combined advanced therapies; in US, the sub-classification covers two major groups of products, i.e., gene therapy and cellular therapy. The inclusion criteria that define a gene therapy are equivalent in both regions, and the exclusion criteria are directly related to the indications of the product. In the EU, there is a clear differentiation between cell- and tissue-based products regarding their classification as advanced therapies or coverage by other legal frameworks, whereas in US, there is a broader classification about whether or not these products can be categorized as biologic products. Both in EU and in US, in order to classify a cell- or a tissue-based product as an advanced therapy, it must be ensured that the processing of the cells implies a manipulation that alters their biological characteristics, although the term of manipulation in US differentiates between structural and non-structural cells and tissues. The regulatory terminology used to define ATMPs and their sub-classification reveals some differences between EU and US.

## Introduction

Advanced therapy medicinal products (ATMPs) comprise a category of innovative and complex biological products, which in most cases require extensive and complicated preclinical and clinical developments. This complexity has been observed since the idea of transferring genetic material to cure a genetic disease was foreseen decades ago. The first ATMP product approved in the European Union (EU) came in 2009 with the authorization of ChondroCelect^®^, a tissue-engineered product indicated for the treatment of cartilage defects (European Medicines Agency, 2017a). In United States (US), the first approved ATMP came out 1 year later with PROVENGE^®^, a somatic cell therapy for the treatment of some prostate cancers (U.S. Food and Drug Administration, 2019a). The first authorized gene therapy was launched in 2012, when Glybera^®^ achieved marketing authorization in EU (European Medicines Agency, 2012).

The delay between the theoretical concept of an ATMP and the first clinical trials that lead to a new treatment approval may be due to the multiple challenges that arise from the nature of ATMPs, including not only scientific and technical challenges but also regulatory ones (Ten Ham et al., 2018). The first step in their development is the definition of the product, and consequently, its classification. In both in EU and the US, there is a broad legal framework, ranging from medicinal products consisting of chemical substances to biological substances; the latter of which includes a wide range of possible products. In this sense, the classification of a potential biological product is often not so trivial, and in some cases, it may be difficult to discern the line between different biological subcategories. The correct classification of a product at an early stage of development is a critical point, since it will determine the regulatory framework and the European and American recommendations to follow throughout the whole development plan of the product in each region.

This article aims to review the legal frameworks in the EU and US for ATMPs, as well as the criteria to be met to define a product as such. The similarities and differences that exist between both regions are discussed in order to identify those nuances that may affect the development of an ATMP. A specific search for official regulatory documents concerning medicinal products for human use with a specific focus on ATMPs, such as legislation, guidelines, presentations, and reports, from the websites of the European Medicines Agency (EMA) and Food and Drug Administration (FDA) competent authorities was carried out until 31st December 2018. Key terms that covered the regulatory framework for advanced therapies and other products were used to navigate the websites of these competent authorities, including: terms describing advanced therapies (advanced therapy, advanced therapies, regenerative medicine, cell therapy, cell-based therapy, human cellular therapy, stem cells, gene therapy, tissue engineering, human cell therapy, human somatic cell therapy), information on the regulatory framework, and the definition and classification of advanced therapies in EU and US.

## Regulatory Framework for the Classification of Advanced Therapies

Medicinal products for human use in EU are governed by Directive 2001/83/EC and Regulation 726/2004/EC. Biological products comprise many diverse product types, including immunological medicinal products (i.e., vaccines, toxins, serums, and allergens), medicinal products derived from human blood and human plasma (i.e., albumin, coagulation factors, and immunoglobulins of human origin), biotechnology products such as antibodies, and ATMPs, which are the focus of this paper (European Union, 2003a). ATMPs consist of products that contain recombinant nucleic acids or engineered cells and/or tissues. These products are divided into four subcategories: somatic cell therapy medicinal products (SCTMP), tissue-engineered products (TEP), gene therapy medicinal products (GTMP), and the combined ATMPs (cATMPs). These last ones consist of one of the first three categories combined with one or more medical devices as an integral part of the product (European Union, 2007). In EU, there is a clear differentiation between cell-based products considered as advanced therapies, and cell-based therapies covered by other legal frameworks such as the blood system or transplant laws, where these cells are not considered a medicinal product, and the active substance, i.e., human cells and tissues, cannot be commercialized or manufactured on an industrial scale for ethical and legal reasons (European Union, 2003b; European Union, 2004; European Union, 2010). The classification of an ATMP as a biological product will determine the wider regulatory framework by which the requirements of the development and the marketing authorization application are defined. These are to be read in conjunction with the specific framework for ATMPs, Regulation 1394/2007/EC, which came into force on December 30, 2008. This regulation provides the overall framework on ATMPs for those products, which are intended to be placed in the market of EU Member States. In addition, Directive 2009/120/EC updated the definitions and detailed scientific and technical requirements for advanced therapies. The cATMPs are not only regulated under the guidelines of medicinal products but also of medical devices. On 25 May 2017, two new regulations on medical devices came into force (European Commission, 2017a).

For the development of advanced therapies in EU, the clinical trial applications are submitted individually to the national competent authorities where the trial will take place. However, for the marketing authorization, all ATMPs are evaluated *via* centralized procedure ensuring that they benefit from a single evaluation and authorization applicable across the EU. There are two committees responsible for the validation and scientific evaluation for product approval: the Committee for Advanced Therapies (CAT) and the Committee for Medicinal Products for Human Use (CHMP) (European Medicines Agency, 2018a). The CAT is the EMA committee responsible for classifying; assessing the quality, safety, and efficacy of ATMPs; and following scientific progress in the field. This committee’s main responsibility is to prepare a draft opinion on each ATMP application submitted to the EMA in order to support the final decision by the CHMP. This marketing authorization *via* the centralized procedure may be granted in three ways: standard marketing authorization, conditional marketing authorization (when an innovative medicine addresses an unmet medical need yet a positive benefit-risk balance by sufficient clinical data is demonstrated), and marketing authorization under exceptional circumstances in those extreme situations where a disease is rare or a clinical endpoint is difficult to measure (Detela and Lodge, 2019). Regarding classification, the CAT offers the confirmation that a medicine meets the scientific criteria to be classified as an ATMP. On the other hand, the regulatory authority in charge of medical devices is the national appointed bodies of each EU member. In the case of cATMP, the CAT interacts with the notified bodies in order to prepare the draft opinion on a cATMP (European Medicines Agency, 2011a).

In US, like in EU, advanced therapies are regulated as biologic products. In legislative terms, biological products comprise the following categories: i) the group of allergenics that includes allergen extracts, allergen patch tests, and antigen skin tests; ii) blood and blood products, iii) vaccines, iv) xenotrasplants, and v) cellular and gene therapy products (CGTs), which constitutes the group of advanced therapies and encompasses two sub-categories of products. Advanced therapies should not be confused with other legislative category of products called “human cells, tissues, and cellular and tissue-based products” (HCT/Ps) and defined as “articles containing or consisting of human cells or tissues intended for implantation, transplantation, infusion, or transfer into a human recipient” (U.S. Food and Drug Administration, 2019). HCT/Ps are not considered biological products. On the other hand, combination products include products that are comprised of two or more regulated components, i.e., drug/device, biologic/device, drug/biologic, or drug/device/biologic. The definition is broad and takes into account the packaging and whether all components of the product are needed to achieve the intended use, indication, or effect (U.S. Food and Drug Administration, 2018a). In 2016, the 21st Century Cures Act (Cures Act) was signed into law in order to help accelerate medicinal product development and bring new therapies to the market faster and more efficiently. This Act established a new expedited product development program called the Regenerative Medicine Advanced Therapy (RMAT) (U.S. Food and Drug Administration, 2018b). Although it is not a type classification *per se*, yet a designation that offers a new expedited option for evaluation of the product, it is considered worth mentioning it here as a part of the US advance therapy classification. A regenerative medicine therapy is defined as: i) a cell therapy, therapeutic tissue-engineering product, human cell and tissue product, or any combination product using such therapies or products, explicitly excluding HCT/Ps; ii) that is intended to treat, modify, reverse, or cure a serious or life-threatening disease or condition; and iii) if the preliminary clinical evidence indicates that the drug has the potential to address unmet medical needs for such disease or condition (U.S. Food and Drug Administration, 2019b). Therefore, this definition implicitly includes advanced therapy medicinal products. A combination product can also be eligible for RMAT designation when the biological product component provides the primary mode of action. These products would be denominated as RMAT-based combination products. More than 30 out of 90 RMAT designation requests have been granted until 2019 (U.S. Food and Drug Administration, 2019c).

The US federal regulatory framework consists of two main statutes, Federal Food, Drug, and Cosmetic Act (FDCA) and the Public Health Services Act (PHSA), which provide the Food and Drug Administration (FDA, the federal regulatory medicines agency in the US) with the legal authority to regulate human medicinal products including drugs, biological products, and devices. Biological products, and therefore advanced therapies, are regulated under section 351 of the PHSA and under the FDCA, because most biological products also meet the definition of “drugs” cited in this Act. FDA regulations are contained in the Code of Federal Regulations (CFR), which provides details on how the FDA implements the activities that are defined in the PHSA and FDCA. Regulations for biological and medical devices are found in Title 21 of the CFR (Lee et al., 2015; U.S. Title 42 The Public Health and Welfare, 2019). In US, the applicants need to submit an investigational new drug (IND) application in order to obtain a clinical trial approval (U.S. Food and Drug Administration, 2017a), and Biologics License Application (BLA) to obtain a marketing authorization (U.S. Food and Drug Administration, 2018c). The marketing authorization can be standard, under a Priority Review procedure or under an Accelerated Approval. In the Priority Review, the application is reviewed within 6 months compared to 10 months under standard review, and it is addressed to those drugs that, if approved, would bring about significant improvements in the safety or effectiveness of the treatment, diagnosis, or prevention of serious conditions when compared to standard applications. An Accelerated Approval allows drugs for serious conditions that filled an unmet medical need to be approved based on a surrogate endpoint, if clinical benefit has been demonstrated (U.S. Food and Drug Administration, 2018d).

Within the FDA, responsibilities for drugs, biologic products and devices are organized in eight different centers. The Centre for Biologics Evaluation and Research (CBER) has jurisdiction over a variety of biological products, including blood and blood products, vaccines and allergenic products, and cellular, tissue, and gene therapies, as well as some related devices. Within the CBER, the responsibility for advanced therapies falls to the Office of Tissues and Advanced Therapies (OTAT), formerly known as Office of Cellular, Tissue, and Gene Therapies (OCTGT). OTAT comprises five divisions in addition to the Office of the Director (U.S. Food and Drug Administration, 2017b). Combination products are assigned to a FDA center that will have primary jurisdiction for its pre-market review and regulation. For combination products, CBER usually regulates medical devices related to licensed blood and cellular products by applying appropriate medical device laws and regulations (U.S. Food and Drug Administration, 2018e). This assignment is performed by the Office of Combination Products through a designation process (U.S. Food and Drug Administration, 2018f).

The current European and American legislations for biological products are summarized in **Table 1**. One of the main differences between EU and US is that the FDA oversees clinical trials, whereas the EMA does not. In terms of marketing approval, each region has specific legislations depending on the legal categorization of the product; in EU, they are licensed under article 8.3 of Directive 2001/83/EC, while in US, ATMPs are licensed under section 351 of the PHS Act. Both Agencies have their own specialized committees to evaluate advanced therapies. In US, the approval time for a standard BLA may extend up to 10 months from receipt date (U.S. Food and Drug Administration 2017c), while in EU, the assessment leads to an opinion from the CHMP by day 210 and European Commission by day 277 (around 7 months) (European Medicines Agency, 2016b). However, these timelines depend on the different types of marketing authorization available in each region. Among advanced therapies, product sub-classifications are slightly different between regions. While in the EU, an ATMP can be sub-classified into four major groups, i.e., GTMP, SCTMP, TEP, or cATMP, in the US the sub-classification groups are broader, covering two groups of products, i.e., gene therapy and cellular therapy products. Given that the sub-classification in the EU is more precise, there are products that could fall into two categories, and in some cases, the assignment in a particular subtype is not so trivial. In the case of US, the difficulty might arise when classifying the product as an HCT/Ps or as a biological product that falls beyond minimal manipulation and/or homologous use. Finally, another difference between regions is related with terminology; in the US, the term “advanced therapy” is not a common term used in legislative and regulatory documents, and these products are collectively referred as “CGT products.”

**Table 1:** Legal and regulatory framework of biological products in United States and European Union.

European Union			United States		
Type of product	Legal framework	Regulatory organism	Type of product	Legal framework	Regulatory organism
**Advanced therapy medicinal products:** Gene therapy products Cell therapy products Tissue-engineered products	Directive 2001/83/EC (relating to medicinal products for human use) Directive 2009/120/EC (relating to medicinal products for human use as regards advanced therapy medicinal products) Regulation 726/2004/EC (community procedures for the authorization and supervision of medicinal products for human and veterinary use and establishing a European Medicines Agency) Regulation 1394/2007/EC (on advanced therapy medicinal products and amending Directive 2001/83/EC and Regulation (EC) No 726/2004)	Clinical trials are under national competent authorities of each member state where the clinical trial will take place. Product positive opinion: CHMP Draft opinion: CAT	**Human somatic cell therapy and gene therapy products**	Section 351 of the PHSA and FDCA and Title 21 CFR 600-680 (Regulation on Biologics) (21 CFR 1271; prevent the spread of infection diseases) RMAT designation: section 3033 of the 21^st^ Century Cures Act (21 U.S.C. 356[g] (8))	CBER and OTAT

CAT, Committee for Advanced Therapies; CBER, Centre for Biologics Evaluation and Research; CHMP, Committee for Human Medicinal Products; FDCA, Federal Food, Drug, and Cosmetic Act; OTAT, Office of Tissues and Advanced Therapies; PHSA, Public Health Services Act; RMAT, Regenerative Medicine Advanced Therapy Designation.

To ensure a correct classification, both the EMA and the FDA have made scientific advice available to the applicants to clarify or corroborate this classification prior to further advancing the development. In EU, one of CAT’s activities is to clarify the classification of a given product, above all when the product could fall in two different categories (European Medicines Agency, 2013a). It is always advisable to obtain CAT’s opinion about a particular product, since the features of each product can be unique, and the corroboration of a product as an advanced therapy might add value to attract potential investors. On the other hand, in US, the Tissue Reference Group is the working group within the FDA that provides recommendations to stakeholders concerning the application of the criteria for HCT/Ps. For both consultations, a minimum of information on the product is required in order to obtain its proper classification, such as the source of the product, the intended use of the product, or description of how the product is processed from the time of recovery to the point of use of step-by-step (U.S. Food and Drug Administration, 2018g). Another consultation option at an early stage of development is to hold informal meetings with the Agencies in order to obtain informal exchange of information and receive advice and recommendations on the development process in terms of scientific, regulatory, and legal issues. For complex products, this type of meeting might also be helpful in order to obtain the first legal and scientific feedback on the classification of the product. For EU, these meetings are called Innovation Task Force (ITF) briefing meetings (European Medicines Agency, 2013a), while the equivalent meeting in US is called Initial Targeted Engagement for Regulatory Advice (INTERACT) meetings (U.S. Food and Drug Administration, 2018h). The ATMP classification procedures are valuable to address questions on borderline classifications, commonly raised for combined ATMPs, to confirm the medicinal product framework and determine what type of ATMP a product is, and therefore, develop the product under the specific dossier requirements and quality guidances.

Finally, it is worth noting that the main EU and US Agencies have launched expedited development programs in order to enable new medicines reach the market as early as possible. The medicines that are eligible to these programs are those that can justify a potential major public health interest, i.e., they target conditions where there is an unmet medical need or have the potential to bring a major therapeutic advantage to patients. Since ATMPs usually offer new treatments for currently incurable conditions or improve existing treatments, most ATMP are eligible to these types of accelerated programs. The FDA has developed the Breakthrough Therapy and Fast Track designation programs (U.S. Food and Drug Administration, 2018d), while the EU launched the adaptive licensing and afterwards the PRIority Medicines (PRIME) designation scheme. The difference between the Breakthrough Therapy and Fast Track designations falls on the qualifying criteria for the designation. In the latter, clinical or nonclinical data should demonstrate potential to address an unmet medical need, whereas in the former, preliminary clinical evidence indicates that it may demonstrate substantial improvement over available therapies on a clinically significant endpoint(s). The EU PRIME and the US Breakthrough Therapy designations share the same objective (timely patient access to innovative medicines) but have a different legal basis; hence, comparison and harmonization are difficult. However, since late 2016, FDA and EMA have worked together to track submitted requests for PRIME and Breakthrough Therapy designations and compare final review outcomes, including specific reasons for a designation request denial (European Medicines Agency, 2018b). Throughout 2019, a database utilizing publicly available and company provided information to create a public list of RMAT recipients, as well as other expedited approval designations awarded in the US, EU, and Japan, is foreseen to be launched (Regulatory Affairs Professional Society, 2019).

## Classification Criteria in Europe and United States

### Gene Therapies

Some examples of gene therapy products include *in vivo* therapies, such as nucleic acids or genetically modified microorganisms (e.g., viruses, bacteria, fungi), and *ex vivo* therapies like genetically modified human cells or human genome editing. In the EU, in order to classify a product as a gene therapy, all of the following inclusion criteria must be met (European Medicines Agency, 2015): i) the product has to be a biological medicinal product according to Directive 2003/63/CE; ii) the product must contain recombinant nucleic acid(s); iii) the recombinant nucleic acids should be of biological origin, regardless of the origin of the vector system used; iv) the recombinant nucleic acid is used in or administered to human beings in order to regulate, repair, replace, add, or delete a genetic sequence; and v) the recombinant nucleic acid(s) should be directly involved in the therapeutic, prophylactic, or diagnostic effect of the product (**Table 2**). It should also be noted that, according to the ATMP Regulation (European Union, 2007), a product that may fall within the definition of a SCTMP or a TEP, and a GTMP, shall be considered a GTMP, since it is the one that can pose the most safety concerns.

**Table 2:** Inclusion/exclusion criteria in European Union.

Advanced Therapy medicinal products				
Product category	Active substance	Purpose	Inclusions	Exclusions
**Gene therapy medicinal products (GTMPs)**	Recombinant nucleic acid of biological origin	Administered to human beings with a view to regulating, repairing, replacing, adding, or deleting a genetic sequence Therapeutic, prophylactic, or diagnostic effects that relate directly to the recombinant nucleic acid sequence it contains, or to the product of genetic expression of this sequence	• Plasmids DNA • Viral vectors • Genetically engineered microorganisms • Human gene–editing technology • Patient-derived cellular gene therapy products	• Non-biological products (e.g., chemical synthetized nucleic acids) • Vaccines against infectious diseases
**Somatic cell therapy medicinal products (SCTMPs)**	Cells or tissues that have been subject to substantial manipulation or not intended to be used for the same essential function(s) in the recipient and the donor	Treating, preventing, or diagnosing a disease through the pharmacological, immunological, or metabolic actions of its cells or tissues	• Products containing or consisting of animal cells or tissues • Cancer immunotherapies • Other autologous and allogeneic cells therapies • Xenogeneic living cells • Stem cells and stem cell–derived products	• Products containing or consisting exclusively of non-viable cells or tissues and which do not act principally by pharmacological, immunological, or metabolic actions
**Tissue-engineered products (TEP)**	Cells or tissues that have been subject to substantial manipulation or not intended to be used for the same essential function(s) in the recipient and the donor The cells or tissues may be viable or non-viable.	Regenerating, repairing, or replacing a human tissue	• Products containing or consisting of animal cells or tissues • Products may also contain additional substances, such as cellular products, bio-molecules, biomaterials, chemical substances, scaffolds or matrices • Products for cartilage or cardiac defects, among others • Stem cells and stem cells-derived products	• Products containing or consisting exclusively of non-viable cells or tissues and which do not act principally by pharmacological, immunological, or metabolic actions
**Combined ATMPs (cATMPs)**	Combines: • one or more medical devices within the meaning of or one or more active implantable medical devices and • its cellular or tissue part must contain viable cells or tissues, or • its cellular or tissue part containing non-viable cells or tissues must be liable to act upon the human body with action that can be considered as primary to that of the devices referred to	Therapeutic, prophylactic, or diagnostic effect Regenerating, repairing, or replacing a human tissue	–	–

In the US, the inclusion criteria that must be met are the following (U.S. Department of Health and Human Services, 1993; U.S. Department of Health and Human Services, 1998): i) the product meets the definition of “biological product” in section 351(i) of the PHSA [42 U.S.C. 262(i)]; ii) the product has to be applicable to the prevention, treatment, or cure of a disease or condition of human beings; iii) the product mediates its effects by transcription or translation of transferred genetic material or by specifically altering host (human) genetic sequences; and iv) the product can work through several mechanisms: replacing a disease-causing gene with a healthy copy of the gene, inactivating a disease-causing gene that is not functioning properly, or introducing a new or modified gene into the body. Recombinant DNA materials used to transfer genetic material for such therapy are considered components of gene therapy (**Table 3**).

**Table 3:** Inclusion/exclusion criteria in United States.

Cell and gene therapy products				
Product category	Definition	Purpose	Examples	Exclusions
**Human gene therapy**	Administration of genetic material to modify or manipulate the expression of a gene product or to alter the biological properties of living cells for therapeutic use	Prevention, treatment, or cure of a disease or condition of human beings	• Plasmid DNA • Viral vectors • Genetically engineered microorganisms • Human gene–editing technology • Patient-derived cellular gene therapy products	• Non-biological products (e.g., chemical synthetized nucleic acids) • Products that are destined for the treatment or prophylaxis of infectious diseases
**Somatic cell therapy**	Autologous, allogeneic, or xenogeneic cells that have been propagated, expanded, selected, pharmacologically treated, or otherwise altered in biological characteristics *ex vivo*	Therapeutic, diagnostic, or preventive purposes	• Cancer vaccines • Cellular immunotherapies • Other types of both autologous and allogeneic cells • Xenogeneic living cells • Stem cells and stem cell–derived products • Gene therapy–modified cells	HCT/Ps under section 361 of the PHSA
**Combination products**				
**Product category**	**Definition**	**Purpose**	**Examples**	**Exclusions**
**Combination products**	Two or more regulated components, i.e., drug, device, biologic as a single entity or packaged together, packaged separately but intended for use only with an approved individually specified drug, device, or biological product where both are required to achieve the intended use, indication, or effect	Therapeutic, diagnostic, or preventive purposes	• Drug/device • Biologic/device: cells combined with medical devices such as natural or synthetic scaffold • Drug/biologic, or • Drug/device/biologic	–
**Regenerative medicine advanced therapy designation**				
**Product category**	**Definition**	**Purpose**	**Examples**	**Exclusions**
Regenerative medicine advanced therapy (RMAT)	A cell therapy, therapeutic tissue-engineering product, human cell and tissue product, or any combination product using such therapies or products	To treat, modify, reverse, or cure a serious or life-threatening disease or condition; To address unmet medical needs for such disease or condition	• AT132 (Audentes Therapeutics, Inc.) • Romyelocel-L (Cellerant Therapeutics, Inc.) • AmnioFix® (MiMedx) • CAP-1002 (Capricor Therapeutics)	Products regulated solely under section 361 of the PHSA are explicitly excluded
**Human cells, tissues, and cellular and tissue-based products**				
**Product category**	**Definition**	**Purpose**	**Examples**	**Exclusions**
HCT/Ps^1^	Articles containing or consisting of human cells or tissues	Implantation, transplantation, infusion, or transfer into a human recipient	• Bone • Ligament • Skin • Dura mate • Heart valve • Cornea • Hematopoietic stem/progenitor cells derived from peripheral and cord blood • Manipulated autologous chondrocytes • Epithelial cells on a synthetic matrix • Semen or other reproductive tissue • Amniotic membrane (when used alone (without added cells) for ocular repair)	• Vascularized human organs for transplantation • Secreted or extracted human products (e.g., milk, collagen, and cell factors) • Minimally manipulated bone marrow for homologous use • Ancillary products used in the manufacture of HCT/P • Cells, tissues, and organs derived from animals other than humans • * In vitro* diagnostic products

^1^HCT/Ps that meet the criteria contemplated in 21 CFR 1721.10(a).

Therefore, despite the different terminology used, the inclusion criteria that define a GTMP are equivalent in both regions: the product must be a biological product that contains “recombinant nucleic acid(s)” (term used in EU) or “genetic material” (term used in US), which through its action mechanism prompts the desired primary effect: addition, manipulation, or modification of gene expressions on human beings. Two autologous chimeric antigen receptor (CAR) T-cell therapies (Kymriah^®^ and Yescarta^®^) were recently approved by the EMA and the FDA. These therapies are classified as cell-based gene therapies in both regions since they consist of genetically modified T cells expressing a CD19-specific CAR in order to lyse CD19-positive targets (normal and malignant B lineage cells). The fact that the product has to be a biological medicinal product is not a minor inclusion criterion, since chemically synthesized nucleic acid sequences will be excluded from being classified as ATMPs and will be considered chemical drugs that should be developed under another legal framework—as for example, antisense oligonucleotides and aptamers approved by the EMA and FDA as chemical drugs. Unlike the US, in EU, one of the inclusion criteria for GTMP establishes that the recombinant nucleic acids should be of biological origin, regardless of the origin of the vector system used. On the other hand, in both regions, the product has to be applicable to the prevention and treatment of a human disease. However, diagnosis is neither cited as one of the primary goals of these products in the US nor does the US definition of a biologic product, according to the PHSA Act, contemplates diagnosis as a purpose of the product (U.S. Title 42 The Public Health and Welfare, 2019). In EU, there is one exclusion criterion that explicitly vetoed a product from being classified as a gene therapy: those products aimed at the treatment or prophylaxis of infectious diseases. These products would be classified as vaccines, even if the product meets all of the necessary criteria to be considered an advanced therapy (European Medicines Agency, 2015). For instance, a modified vaccinia virus ankara (MVA) into which two genes have been placed for the treatment of non-small cell lung cancer is classified as a GTMP, but if these genes lead to foreign protein expression for the treatment of human immunodeficiency virus (HIV) disease, the product will not be considered an advanced therapy, but a vaccine (European Medicines Agency, 2016b; Draper and Heeney, 2010). The same principle applies to non-viral vectored products such as most plasmid DNA- or RNA-based products. For instance, Trimix is a mixture of mRNAs encoding for antigen presenting cell activation molecules. If this mixture of mRNAs is combined with tumor-associated antigens for the treatment of melanoma, the therapy is classified as a GTMP, but if these mRNA are combined with mRNA encoding for HIV antigens, the therapy will be considered a vaccine (European Medicines Agency, 2016b). In the US, it is not specifically mentioned as an exclusion criterion, but prophylaxis or therapeutic vaccines for infectious diseases have their own guidances for development, and these products are typically reviewed by the CBER/Office of Vaccines Research and Review (OVRR) and not by the OTAT (U.S. Department of Health and Human Services, 2007). Therefore, the criterion for excluding a product from being classified as a GTMP in both regions is directly related to the indications of the product. Although some regulatory and development requirements for both types of products overlap, since these vaccines may be gene-based, for either region, there are guidelines specifically addressed to the development of vaccines or gene therapy products independently. A consequence of this classification is that some of the available EU regulatory procedures that facilitate the development of ATMPs would not apply in the case of products classified as vaccines; for instance, the possibility of certifying the quality and non-clinical data for ATMP applications by the EMA (European Medicines Agency, 2010).

### Cell and Tissue Therapies

In the EU, SCTMP are distinguished from TEP. However, both class products share the same inclusion principle, i.e., the cells or tissues of the product must be “engineered,” and the difference lays in the indication. To consider a cell or tissue as “engineered,” it must fulfill at least one of the following criteria (European Union, 2007): i) the cells or tissues have been subject to substantial manipulation, or ii) the cells or tissues are not intended to be used for the same essential function(s) in the recipient and the donor, i.e., non-homologous use. Regarding the indication, in the case of SCTMP, the product is administered to human beings with a view to treating, preventing, or diagnosing a disease through the pharmacological, immunological, or metabolic actions of its cells or tissues, whereas in the case of TEP, the product is administered to human beings with a view to regenerating, repairing, or replacing human tissue. The key to ascertain the most appropriate subcategory is based on the predominant mechanism of action of the active substance and the claimed intended function. A problem arises when the dividing line for classifying a product as SCTMPs or TEP is not clear. Such is the case when the product exerts a pharmacological action in order to regenerate, repair, or replace a human tissue. For these cases, premises have been established in order to categorize a specific product: a product which may fall within the definition of a TEP and SCTMP should be considered a TEP according to ATMP Regulation, although the final classification should be considered on case-by-case basis, playing CAT’s opinion a major role. In addition, those products that consist of engineered or manipulated cells that induce regeneration, repair, or replacement in the native tissue *via* secretion of paracrine factors also fulfill the definition of a TEP (European Medicines Agency, 2015). Finally, it is considered that a TEP may contain cells or tissues of human or animal origin, or both, and that the cells or tissues may be viable or non-viable, considering viable cells those that have a functional cytoplasmic membrane. Two considerations in this regard are made: i) an inclusion criterion that automatically classifies a product as an ATMP applies when products contain or consist of animal cells or tissues and ii) an exclusion criterion for not classifying a potential product either as a SCTMP or TEP includes those products containing or consisting exclusively of non-viable cells or tissues and which do not act principally through pharmacological, immunological, or metabolic actions (**Table 2**).

As mentioned, cell- and tissue-based products can be sub-categorized in the US regulatory framework as biologic products or as HCT/Ps. The definition of cell- and tissue-based products regulated as biologic products includes those that are “more-than-minimally manipulated,” or for “non-homologous use,” or have a systemic effect, or depend on its metabolic activity (except for autologous cells, allogeneic cells for 1st of 2nd degree relatives and reproductive cells) (U.S. Department of Health and Human Services, 2017a). The group of advanced therapies referred to as “human somatic cell therapy products” fall within this definition. Note that, in US, there is no product class defined for tissue-based advanced therapies. The definition and the inclusion criteria for human somatic cell therapy (SCT) include the following (U.S. Department of Health and Human Services, 1993; U.S. Department of Health and Human Services, 1998): i) SCT consists of administration to humans of autologous, allogeneic, or xenogeneic living cells; ii) the manufacture of products for SCT involves the *ex vivo* propagation, expansion, selection or pharmacologic treatment of cells, or other alterations of their biological characteristics, and therefore considered “more-than-minimally manipulated”; and iii) the aim of this cellular products is to be used for therapeutic, diagnostic, or preventive purposes (**Table 3**).

Therefore, the categorization or classification of human cells and tissue products between the EU and the US is different. On one hand, in the EU, there is a differentiation between products considered TEP, or SCTMP, in which the difference lies in the claimed indication, while in the US, cell and tissue products that constitute an advanced therapy will be labeled under the SCT’s term. For instance, MACI (matrix-applied characterized autologous cultured chondrocytes) is a product approved both in EU and US which consists of autologous chondrocytes seeded on a collagen membrane of porcine origin indicated for the repair of symptomatic, full-thickness cartilage defects of the knee in adult patients. While in US, MACI is considered a cell therapy, a biologic-device combination product with the aim of being used for therapeutic purposes; in EU, it is classified as combined TEP, since the claimed primary mechanism of action of the product is the regeneration, repair, and replacement actions (European Medicines Agency, 2018d; U.S. Food and Drug Administration, 2018i). Finally, the FDA classifies xenogeneic living cells as SCT, as well as in EU, where these therapies can be assumed to be automatically classified as ATMPs from a regulatory point of view (European Medicines Agency, 2009; Schuurman, 2015).

#### Manipulation and Homologous Use

Both aforementioned inclusion criteria, manipulation and homologous use, have their own definitions depending on the region. In EU, “substantial manipulation” means to modify the biological characteristics, physiological functions, or structural properties relevant for the intended clinical use. For instance, cell separation, concentration, or purification does not represent a substantial manipulation if the cells performed the same biological activity as in the human body, whereas cell-culturing leading to expansion or cell activation with growth factors does. A non-exhaustive list of manipulations that is not considered substantial for ATMP purposes is provided in Annex I of Regulation EC (No). 1394/2007 and includes: cutting, grinding, shaping, centrifugation, soaking in antibiotic or antimicrobial solutions, sterilization, irradiation, cell separation, concentration, or purification, filtration, lyophilization, freezing, and cryopreservation. On the other hand, the “same essential function” (or homologous use) means that the cells or tissues (whether substantially manipulated or not) are used to maintain the original function(s) in the same anatomical or histological environment. By contrast, “different essential function” (or non-homologous use) for cells or tissues (substantially manipulated or not) are those not intended to be used for the same essential function(s) in the recipient as the original cell/tissue would perform in the donor (European Medicines Agency, 2015). Allogeneic human islets of Langerhans for the treatment of severe forms of type 1 diabetes is a common example of cell/tissue products that might be regarded as non-ATMPs, since these cells/tissues might be isolated, purified, and cultured by methods that do not result in a modification of the biological characteristics and are re-administered to fulfill their same essential function. In 2011, CAT considered that autologous/allogeneic human islets of Langerhans were not an ATMP (European Medicines Agency, 2011b), but are considered to fall under the provisions of the Tissues and Cells legislation. Under this legislation, these cells are neither considered a medicinal product, since the active substance, i.e., human tissues, cannot be commercialized or manufactured on an industrial scale for ethical and legal reasons. However, in 2013, a product that consists of viable alginate encapsulated porcine pancreatic islet cells was classified as a SCTMP (European Medicines Agency, 2013b). In this case, the porcine islets were isolated from pancreases of neonatal piglets and cultured during 30 days, in which cell differentiation occurs by increasing the amount of insulin released from the cells, this being considered a substantial manipulation. Nevertheless, it should be noted that, since this product is based on xenogeneic cells, it is automatically considered an ATMP, as previously discussed. Finally, in 2018, the CAT considered an encapsulated allogeneic pancreatic islet–based product a non-ATMP. The consideration here is whether or not the encapsulation itself might change the characteristics of the islet (European Medicines Agency, 2018e).

In US, the definitions of manipulation and homologous use are defined for HCT/Ps, and by exclusion, the products based on cells and tissues that do not comply with these criteria established for a HTC/Ps could be considered a biological product, and consequently, an advanced therapy (U.S. Department of Health and Human Services, 2017a). The criteria for HCT/Ps include “minimal manipulation” and “homologous use,” while “more-than-minimally manipulated” and “non-homologous use” are considered for cell- and tissue-based products considered as biological drugs.

Unlike the EU, in the US, there is a differential definition of minimal manipulation depending on whether or not the product consists of structural tissue. “Minimal manipulation” is defined as: “processing that does not alter the original relevant characteristics of the tissue relating to the tissue’s utility for reconstruction, repair, or replacement” for structural tissues, and “processing that does not alter the relevant biological characteristics of cells or tissues” for cells or non-structural tissues (U.S. Code of Federal Regulation Title 21, 2018a). For clarification, structural tissue is defined as human cells/tissues that physically support or serve as a barrier or conduit, or connect, cover, or cushion (e.g., amniotic membrane and umbilical cord). On the other hand, human cells/tissues that serve as metabolic or other biochemical roles in the body, such as hematopoietic, immune, and endocrine functions, are generally considered cells/non-structural tissues (e.g., hematopoietic stem/progenitor cells). It is considered that this differentiation between structural and non-structural tissues is required, since structural HCT/Ps generally raise different safety and efficacy concerns from those of cells or non-structural tissues.

As a result, the term “processing” is defined as any activity performed on a cell- and/or tissue-based product other than recovery, donor screening, donor testing, storage, labeling, packaging, or distribution, such as testing for microorganisms, preparation, sterilization, steps to inactivate or remove adventitious agents, preservation for storage, and removal from storage. Processing includes cutting, grinding, shaping, culturing, enzymatic digestion, and decellularization (U.S. Code of Federal Regulation Title 21, 2018a). Cell expansion, encapsulation, activation, or genetic modification are considered to be more than minimal manipulations. The aforementioned or any other additional processing steps should be considered in determining whether a product is minimally manipulated or not.

For products that contain structural tissues, “original relevant characteristics of structural tissues” generally comprise the properties of that tissue in the donor that contribute to the tissue’s function or functions; for instance, the original relevant characteristics of amniotic membrane generally include the physical integrity, tensile strength, and elasticity of the tissue. Following with the same example, preserving and packaging amniotic membrane in sheets would be considered a minimal manipulation, yet more than minimally manipulated if the amniotic membrane is grounded, lyophilized, and packaged as particles, since it would imply the separation of structural tissue into components whose characteristics related to serving as a barrier are altered. However, ground bone adhered to form bone particles would generally be considered minimally manipulated since it can maintain its utility as a supporting structure. For products that contain cells (both structural and non-structural) and non-structural tissues, “original relevant characteristics” include differentiation and activation state, proliferation potential, and metabolic activity, e.g., for hematopoietic stem/progenitor cells, the ability to repopulate the bone marrow by self-renewal and by differentiating along myeloid and lymphoid cell lines. In this case, cell selection on peripheral blood apheresis products to obtain a higher concentration of hematopoietic stem/progenitor cells for transplantation would be considered a minimal manipulation, whereas differentiating the cells by culturing under specific conditions would be considered more than a minimal manipulation because the characteristics of multipotency and capacity for self-renewal are altered. The storage of the product should also be considered, since it can alter the original relevant characteristics of the cells and tissues. If a product is stored in a buffer solution or is cryopreserved, it would generally meet the minimal manipulation criterion.

Regarding “homologous use,” there is also a differentiation between structural and non-structural tissues. The term of homologous use for a structural tissue defines that the tissue is intended to be used for a homologous function when used to replace an analogous structural tissue that has been damaged or otherwise does not function adequately. Therefore, it is defined as the repair, reconstruction, replacement, or supplementation of a recipient’s cells or tissues with an HCT/P that performs the same basic function or functions in the recipient as in the donor (U.S. Code of Federal Regulation Title 21, 2018a). The Agency would consider structural tissue to be performing a non-homologous function when used for a purpose different from those that it fulfils in its native state, or in a location of the body, where such structural function does not normally occur. Similarly, cellular products are considered to be used for a homologous function when they are used to perform their native function, and for a non-homologous function when they are used to perform other functions (U.S. Department of Health and Human Services, 2017a; U.S. Department of Health and Human Services, 2017b).

As it has been discussed, it is important to have a product defined since, otherwise, the legal requirements for these could be violated. In the US, this was the case of some amniotic-/chorionic-based products, used for wound healing, which were considered HCT/Ps by some companies, when in fact, they were biological products. These products were therefore launched to the market without a premarket review, and after an inspection of the CBER Office of Compliance and Biologics Quality, an appropriate clinical development was requested in order to demonstrate the safety and efficacy of the intended use of the product, as well as distribution of the product to test its clinical use in humans after IND application, and the subsequent submission of a BLA approval for its marketing. This implied that the cost of bringing this product to the market was very different from the one initially invested, given that the preclinical and clinical developments are much broader than for an HCT/Ps (U.S. Food and Drug Administration, 2018j; U.S. Food and Drug Administration, 2018k).

Therefore, both in the EU and in the US, in order to consider a cell- and tissue-based products advanced therapies, it must be ascertained that the processing of the cells implies a manipulation that alters their biological characteristics. In EU, the concept is referred as a “substantial manipulation,” while in US, it is referred as “more-than-minimally manipulated.” Regarding this term of manipulation in US, there is a nuance that differs from EU definitions and consists in the differentiation of structural and cells/non-structural tissues in the US. The European definitions of substantial manipulation and non-homologous use would encompass both structural and non-structural tissues under the same definition. Regardless of the examples of processing mentioned for either regions, for both, it is key to determine if the processing changes the original characteristics of the product. This requires a characterization of the product during the manufacturing process, as a part of development, to corroborate whether or not the phenotypic and physiological characteristics of a potential product have been altered. On the other hand, the European terminology uses the term “engineered” to denominate those cells or tissues that are substantially manipulated and/or used for a different essential function (or non-homologous use), which is mandatory criteria to classify a product as an advanced therapy. In the US, the term of non-homologous use is not explicitly mentioned in the definition of SCT, although it is to classify a product as biologic in the general definition of cell- and tissue-based products. Note that the nomenclature of “non-homologous use” is be common for both regions, although in Europe, the term “different essential function” would also be the one harmonized according to the EMA guidelines. All these mentioned differences in terminology can be important when submitting documents to the respective Agencies, since it is advisable to use the specific terminology used in each region (**Table 4**).

**Table 4:** Terminology and definitions for cell- and tissue-based products as advanced therapies.

European Union^1^		United States^2^	
**Term**	Definition	Term	Definition
**Substantial manipulation**	Biological characteristics, physiological functions, or structural properties have been modified to be relevant for their intended function during the manufacturing process.	More than “minimally manipulated”*	For structural tissue, processing that alters the original relevant characteristics of the tissue relating to the tissue’s utility for reconstruction, repair, or replacement
			For cells or non-structural tissues, processing that alters the relevant biological characteristics of cells or tissues
**Different essential function or non-homologous use**	Cells when removed from their original environment in the human body are not used to maintain the original function(s) in the same anatomical or histological environment.	**Non-homologous use**	Homologous use means the repair, reconstruction, replacement, or supplementation of a recipient’s cells or tissues with an HCT/P that performs the same basic function or functions in the recipient as in the donor, including when such cells or tissues are for autologous use.
			Basic functions of a structural tissue would generally be to perform a structural function for example, to physically support or serve as a barrier or conduit, or connect, cover, or cushion. Basic functions of a cellular or nonstructural tissue would generally be a metabolic or biochemical function, such as hematopoietic, immune, and endocrine functions.
**Manufacturing**	Defined to include all operations of receipt of materials, production, packaging, repackaging, labeling, relabeling, quality control, release, storage, and distribution of active substance(s) and the related controls	**Processing**	Any activity performed on an cell- and/or tissue-based product, other than recovery, donor screening, donor testing, storage, labeling, packaging, or distribution, such as testing for microorganisms, preparation, sterilization, steps to inactivate or remove adventitious agents, preservation for storage, and removal from storage
**List of manipulations**	Provided in Annex I of Regulation EC (No.) 1394/2007	**List of processing**	Provided in regulatory considerations for human cells, tissues, and cellular and tissue-based products: minimal manipulation and homologous use (2017) and proposed approach to regulation of cellular and tissue-based products (1997), and the United States pharmacopoeia (cellular and tissue-based products: 1046)
**–**	–	**Original relevant characteristics**	For products that contain structural tissues, “original relevant characteristics of structural tissues” generally include the properties of that tissue in the donor that contribute to the tissue’s function or functions.
			For products that contain cells (both structural and non-structural) and non-structural tissues, “original relevant characteristics” include differentiation and activation state, proliferation potential, and metabolic activity.
**Viable cell**	A viable cell is a cell that has a functional cytoplasmic membrane. (The European Pharmacopoeia provides information on assays to demonstrate cytoplasmic membrane integrity and activity < 20729 >.)	**Living cells**	– (The United States pharmacopoeia cellular and tissue-based products <1046>)
**Tissues**	Defined in Directive 2004/23/EC (Art 3.b) as “all constituent parts of a human body formed by cells.”	**–**	–

^1^Definitions provided in EMA/CAT/600280/2010 Rev.1, CPMP/ICH/4106/00 and Regulation EC (No) 1394/2007; ^2^Definitions provided in the Code of Federal Regulation (21 CFR 1271.3; 21 CFR 1271.10), Regulatory Considerations for Human Cells, Tissues, and Cellular and Tissue-Based Products: Minimal Manipulation and Homologous Use (2017) and Proposed approach to regulation of cellular and tissue-based products (1997); *The definition provided is minimal manipulation. For advanced therapies the term that applies is “more than minimally manipulated”.

### Combined Advanced Therapy Medicinal Products

In EU, there is a specific category for those products that consist in an ATMP combined with a medical device. A medical device is defined as any instrument, apparatus, appliance, material, or other article intended by the manufacturer to be used on human beings for the purpose of: i) diagnosis, prevention, monitoring, treatment or alleviation of disease, compensation for an injury or handicap, investigation, replacement, or modification of the anatomy of a physiological process, or control of conception and ii) which does not achieve its principal intended action in or on the human body by pharmacological, immunological, or metabolic means, but may assist its function by such means (European Union, 2017). Examples of medical devices in cATMP could be scaffolds, matrices, and encapsulation systems for cells, such as microspheres, among others. The criteria to meet in this category class are that the product must incorporate, as an integral part of the product, one or more medical devices. The medical device should be used in the combination, in the same way as its intended use without additional components. On the other hand, the cellular or tissue part of the product must contain viable cells or tissues, or if containing non-viable cells or tissues, it must be liable to act upon the human body with actions that can be considered primary to those of the devices referred to (European Medicines Agency, 2015).

In US, there is no specific category for cATMPs, but there are nine different types of combined products including drug/device, biologic/device, drug/biologic, or drug/device/biologic. The definition takes into account how the product is packaged, i.e., together in a single package or packaged separately, and if all components of the product are needed to achieve the intended use, indication, or effect. Among all of these categories, the type-5 combination product named “device coated or otherwise combined with biologic” constitutes the biologic/device combination where the device has an additional function in addition to delivering the drug and constitutes an “integral part” of the final product, e.g., live cells seeded on or in a device scaffold (U.S. Food and Drug Administration, 2018a). In US, a medical device is defined as an instrument, apparatus, implement, machine, contrivance, implant, *in vitro* reagent, or other similar or related article, including any component, part, or accessory, which has at least one of the following three characteristics: i) it is recognized in the official National Formulary or the United States Pharmacopeia, or any supplement to them; ii) it is intended for use in the diagnosis, cure, mitigation, treatment, or prevention of a disease; or iii) is intended to affect the structure or any function of the human body or other animals and does not achieve its primary intended purposes through chemical action within or on the human body or other animals and which does not depend on being metabolized for the achievement of its primary intended purposes (U.S. Code of Federal Regulation Title 21, 2018b; U.S. Food and Drug Administration, 2018l).

Therefore, while in EU, cATMPs are the fourth subcategory of products within the group of advanced therapies; in the US, the subcategory defined for combined products is very broad and includes drugs, biological, and medical devices. The category of type-5 combined products would constitute a group equivalent to what defines cATMPs in the US, where the product is a single-entity combination product, or the device constitutes “an integral part of the product” according to European definition. For both EU and US, the final combined product will be a biological and a medical device, where the definitions of medical device are equivalent: the medical device assists in the primary function of the biological component. Following MACI’s aforementioned example, for both regions, the porcine collagen membrane is considered a device constituent of the product a CE-marked class III device in EU (European Medicines Agency, 2018d; U.S. Food and Drug Administration, 2018i).

The fact of combining a biological product with a medical product complicates its development, and in US, unlike in EU, there are guidances with some considerations to be taken into account during the development of these products (U.S. Food and Drug Administration, 2006; U.S. Food and Drug Administration, 2019d).

## General Discussion and Conclusion

Our analysis reveals a difference between EU and US in the sub-categorization of advanced therapies and the regulatory terminology defining them. The criteria that must be met in both the EU and the US in order to classify a product as an advanced therapy is similar, although EU presents a more precise sub-classification with more defined inclusion criteria between these subcategories. The criteria to determine if a product qualifies as a gene therapy may be simpler or more obvious than for cell therapies, although there are some relevant considerations for all defined subcategories of advanced therapies that can change the classification of the product both in EU and US.

EU and US are facing similar challenges regarding the regulation of ATMPs due to their inexperience in this specific medicinal product group, and because Europe covers a variety of overlaying jurisdictions and authorities on a member state level (Bender, 2018; Ten Ham et al., 2018). European and American legislation and regulatory guidelines launched by EMA and FDA show similarities and differences in the ATMP classification for both regions. It is unknown if these differences can be translated into divergent final recommendations by the regulatory authorities. Currently, the number and type of ATMPs approved differ between the two regulatory Agencies. In EU, up to 12 ATMPs have been authorized from 2009, but four of them have been withdrawn throughout the past 10 years. In US, nine gene and cell therapies haven’t been authorized, and only six of them match in the two Agencies. The rationale behind these differences is unknown, but it seems feasible that a worldwide harmonization of the procedures involved in the development of ATMPs may allow to reach similar ultimate decisions. It is acknowledged that EMA and FDA have been collaborating for the past 15 years with the aim to ameliorate regulatory excellence. An ATMP cluster has been created under the umbrella of the reinforced EU/US collaboration on medicines with the aim to facilitate regulatory excellence of the new medicinal products (European Medicines Agency, 2018f). Yet, Agencies’ recommendations are evolving and being updated over time in a non-parallel manner. In 2018, the FDA launched several guidances that include specific recommendations for the development of ATMPs aimed at certain types of diseases such as hemophilia or retinal disorders (U.S. Food and Drug Administration, 2019e), while the EMA guidelines published to date are more generalist, encompassing only the development of ATMPs according to the three main groups of therapies, GTMP, SCTMP, and TEP. In the future, it would be convenient to begin a progressive process of convergence between both Agencies in terms of terminology, legal recommendations, and characterization requirements. In this regard, some steps could be taken to reach this alignment between regulators—for example, common guidelines, increased number of EMA/FDA parallel scientific advice from the beginning of the lifecycle of the medicinal product, as well as similar post-authorization monitoring of the products or real-world evidence data generation.

## Author Contributions

CIL conducted the bibliographic search, analysed the data, drafted the manuscript and edits the final copy of this article. AA, MO, and AV analyzed and reviewed the data and edited the manuscript.

## Conflict of Interest Statement

The authors declare that the research was conducted in the absence of any commercial or financial relationships that could be construed as a potential conflict of interest.
