# Corrigendum: Regulatory Framework for Advanced Therapy Medicinal Products in Europe and United States

**DOI:** 10.3389/fphar.2020.00766

**Published:** 2020-05-25

**Authors:** Carolina Iglesias-Lopez, Antonia Agustí, Mercè Obach, Antonio Vallano

**Affiliations:** ^1^Department of Pharmacology, Therapeutics and Toxicology, Universitat Autònoma de Barcelona, Barcelona, Spain; ^2^Clinical Pharmacology Service, Vall d'Hebron University Hospital, Barcelona, Spain; ^3^Medicines Department, Catalan Healthcare Service, Barcelona, Spain; ^4^Pathology and Experimental Therapeutics Department, University of Barcelona, Barcelona, Spain

**Keywords:** genetic therapy, tissue engineering, cell- and tissue-based therapy, biological products, biological therapy, legislation and jurisprudence, United States Food and Drug Administration, Europe

In the original article, there was an error. In the specified paragraph, the word “latter” and “former” should be interchanged. A correction has been made to the **Regulatory Framework for the Classification of Advanced Therapies** section, paragraph 8:

“Finally, it is worth noting that the main EU and US Agencies have launched expedited development programs in order to enable new medicines reach the market as early as possible. The medicines that are eligible to these programs are those that can justify a potential major public health interest, i.e., they target conditions where there is an unmet medical need or have the potential to bring a major therapeutic advantage to patients. Since ATMPs usually offer new treatments for currently incurable conditions or improve existing treatments, most ATMP are eligible to these types of accelerated programs. The FDA has developed the Breakthrough Therapy and Fast Track designation programs (U.S. Food and Drug Administration, 2018d), while the EU launched the adaptive licensing and afterwards the PRIority Medicines (PRIME) designation scheme. The difference between the Breakthrough Therapy and Fast track designations falls on the qualifying criteria for the designation. In the latter, clinical or nonclinical data should demonstrate potential to address an unmet medical need, whereas in the former, preliminary clinical evidence indicates that it may demonstrate substantial improvement over available therapies on a clinically significant endpoint(s). The EU PRIME and the US Breakthrough Therapy designations share the same objective (timely patient access to innovative medicines) but have a different legal basis; hence, comparison and harmonization are difficult. However, since late 2016, FDA and EMA have worked together to track submitted requests for PRIME and Breakthrough Therapy designations and compare final review outcomes, including specific reasons for a designation request denial (European Medicines Agency, 2018b). Throughout 2019, a database utilizing publicly available and company provided information to create a public list of RMAT recipients, as well as other expedited approval designations awarded in the US, EU, and Japan, is foreseen to be launched (Regulatory Affairs Professional Society, 2019).”

The authors apologize for this error and state that this does not change the scientific conclusions of the article in any way. The original article has been updated.
